# Clinical Modelling of RVHF Using Pre-Operative Variables: A Direct and Inverse Feature Extraction Technique

**DOI:** 10.3390/diagnostics12123061

**Published:** 2022-12-06

**Authors:** Dilber Uzun Ozsahin, Ozlem Balcioglu, Abdullahi Garba Usman, Declan Ikechukwu Emegano, Berna Uzun, Sani Isah Abba, Ilker Ozsahin, Tahir Yagdi, Cagatay Engin

**Affiliations:** 1 Medical Diagnostic Imaging Department, College of Health Science, University of Sharjah, Sharjah 27272, United Arab Emirates; 2 Operational Research Centre in Healthcare, Near East University, TRNC Mersin 10, Nicosia 99138, Turkey; 3 Department of Cardiovascular Surgery, Near East University, TRNC Mersin 10, Nicosia 99138, Turkey; 4 Statistics Department, Carlos III University of Madrid, 28903 Madrid, Spain; 5 Department of Mathematics, Near East University, TRNC Mersin 10, Nicosia 99138, Turkey; 6 Interdisciplinary Research Center for Membrane and Water Security, King Fahd University of Petroleum and Minerals, Dhahran 31261, Saudi Arabia; 7 Department of Radiology, Brain Health Imaging Institute, Weill Cornell Medicine, New York, NY 10065, USA; 8 Cardiovascular Surgery Department, Ege University, Izmir 35100, Turkey

**Keywords:** right ventricular heart failure, pre-operative, artificial intelligence, hybrid-based paradigms, validation step

## Abstract

Right ventricular heart failure (RVHF) mostly occurs due to the failure of the left-side of the heart. RVHF is a serious disease that leads to swelling of the abdomen, ankles, liver, kidneys, and gastrointestinal (GI) tract. A total of 506 heart-failure subjects from the Faculty of Medicine, Cardiovascular Surgery Department, Ege University, Turkey, who suffered from a severe heart failure and are currently receiving support from a ventricular assistance device, were involved in the current study. Therefore, the current study explored the application of both the direct and inverse modelling approaches, based on the correlation analysis feature extraction performance of various pre-operative variables of the subjects, for the prediction of RVHF. The study equally employs both single and hybrid paradigms for the prediction of RVHF using different pre-operative variables. The visualized and quantitative performance of the direct and inverse modelling approach indicates the robust prediction performance of the hybrid paradigms over the single techniques in both the calibration and validation steps. Whereby, the quantitative performance of the hybrid techniques, based on the Nash–Sutcliffe coefficient (NC) metric, depicts its superiority over the single paradigms by up to 58.7%/75.5% and 80.3%/51% for the calibration/validation phases in the direct and inverse modelling approaches, respectively. Moreover, to the best knowledge of the authors, this is the first study to report the implementation of direct and inverse modelling on clinical data. The findings of the current study indicates the possibility of applying these novel hybridised paradigms for the prediction of RVHF using pre-operative variables.

## 1. Introduction

Right ventricular heart failure (RHVF) is generally due to problems arising from the left atrium [1]. This heart disease is a syndrome characterised by the inability of the cardiac output to match the body’s metabolic demands, stemming from structural or functional impairment of ventricular filling or ejection. There are, however, two predominant conditions where the anatomic right ventricular (RV) lies in the systemic position and the anatomic left ventricular (LV) lies in the subpulmonary position, namely d-Transposition of the great arteries (d-TGA), which is palliated with an atrial switch operation, and congenitally corrected transposition of the great arteries (cc-TGA) [2]

Most patients with symptoms and signs of heart failure have a left ventricular ejection fraction that is not markedly abnormal [3]. The recognition of the magnitude of the problem of heart failure with preserved ejection fraction in the past 20 years has spurred an explosion of clinical investigation and a growing intensity of informative outcome trials [4].

Although substantial progress has been made in understanding the various pathological conditions of RVHF, effective and accurate medical assessment of these factors, as well as the benefits of RVHF diagnosis at an earlier stage, have led to a tremendous surge in the use of detectors [5,6,7]. Furthermore, Manca et al. [8] reported that Titin (TTN)-related dilated cardiomyopathy (DCM) has a higher likelihood of left ventricular reverse remodelling compared with other genetic etiologies. Moreover, research indicates that previously there were no data regarding the evolution of right ventricular dysfunction (RVD) according to genetic background. Moreover, the findings of these results indicate that the evolution of RVD in DCM is heterogeneous in different genetic backgrounds. Whereby, TTN-related DCM is associated with a higher chance of RVD recovery compared with other genetic etiologies.

Therefore, the need for understanding this medical condition using a simple, fast, and cost-effective technique is of paramount importance. For instance, the application of artificial intelligence (AI) and machine learning (ML) in locating, analysing, interpreting, forecasting, and classifying the medical information associated with RVHF can serve as the robust integration for understanding this important health condition, especially when provided with the necessary medical information. Recently, ML and AI have been recommended as valuable methods to enhance illness prognosis, diagnosis, and prediction as well as progress management [5,9]. Several machine learning (ML) algorithms have been implemented in RVHF to improve the medical workflow and avoid the limitations of conventional methods. A recent example is the ML-based research by Jingjing et al. [10], which uses AI-assisted auscultation to detect congenital cardiac disorders at a Shanghai children’s medical center; they focused on the sensitivity, specificity, and accuracy of remote auscultation. This study found that remote auscultation could find unusual heart sounds with 98% responsiveness, 91% specificity, 97% accuracy, and a 0.87 kappa coefficient. A total of 1397 people with CHD signed up for the survey. The rest of the specimens from the 1362-patient population (mean age 2.4 3.1 years, 46% female) were evaluated.

Silvia et al. [11] reported that cardiovascular disease (CVD), despite the significant advances in its diagnosis and treatment, still represents the leading cause of morbidity and mortality worldwide. Therefore, to improve and optimise CVD outcomes, artificial intelligence techniques have the potential to radically change the way we practice cardiology, offering us novel tools to interpret data and make clinical decisions. AI techniques such as machine learning and deep learning can also improve medical knowledge due to the increase in the volume and complexity of the data, unlocking clinically relevant information.

**Figure 1:**
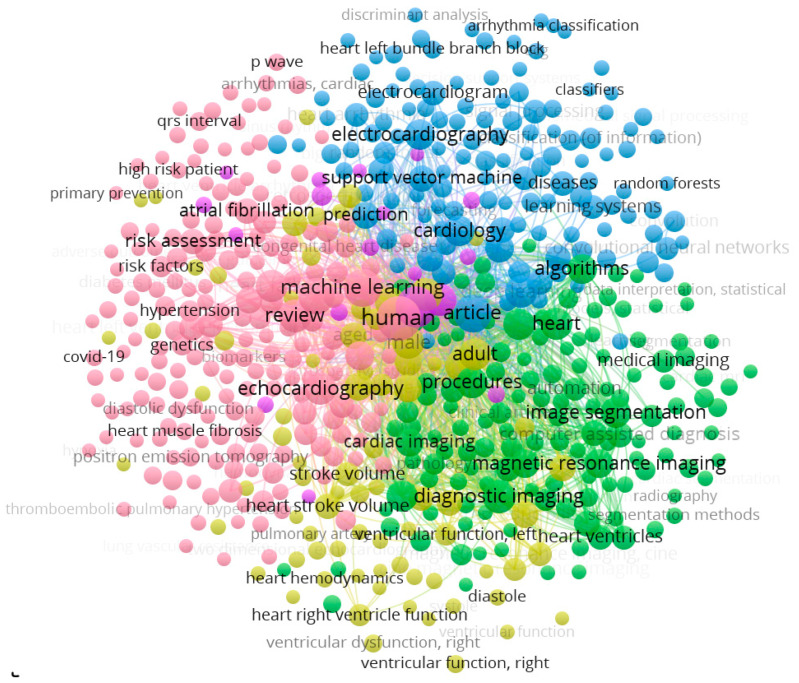
Biblographic literature scan from 1980–2022 on keywords, titles, and abstracts related to implementation of machine learning on RVHF data (source: Scopus 2022).

The current study is, to our best knowledge, the first in the technical published literature that employs the applications of hybridised paradigms (ILR–ANFS, ILR–GPR, and ILR–GRNN) for the clinical prediction of RVHF using pre-operative variables and, ultimately, is based on the recent technical literature as well as a scan of the literature, as shown in Figure 1; in addition, to our best knowledge, this is the first study that reports the feasibility of applying direct and inverse modelling for clinical prediction in health- and medical-related studies.

Therefore, the current study aims at understanding the connection between pre-operative variables and RVHF by using the correlational feature extraction method based on heart failure patients’ data. Based on the correlational analysis results of the pre-operative variables and RVHF as the dependent variable, two novel approaches were developed, namely the inverse and direct approaches for modelling RVHF using single stand-alone models with improved novel hybridised paradigms.

## 2. Materials and Methods

### 2.1. Single Paradigms

#### 2.1.1. Gaussian Process Regression (GPR)

Gaussian process regression (GPR) can be referred to as robust non-linear prediction model, which is probabilistic, nonparametric, supervised, and unsupervised learning method that generalises the non-linear and complex function mapping hidden in datasets. Recently, GPR has increasingly attracted the attention of researchers from different engineering fields [12,13]. GPR is capable of handling non-linear data due to its use of kernel functions. Moreover, one of the merits of a GPR model is that the model can provide a reliable response to input data [14].

#### 2.1.2. Adaptive Neuro-Fuzzy Inference System (ANFIS)

When there are problems, ANFIS can figure out what they are and how to fix them. Its origin was from the feed-forward and multilayer adaptive networks. ANFIS is jointly made up of input variables and the rule of fuzzy, which has dependent and independent variables according to TSK (Takagi–Sugeno–Kan) inferences. Fuzzification and defuzzier are all contained in the database of fuzzy. By using membership function parameters, fuzzy set theory converts the information into fuzzified values.

Nodes had a vital role as member functions (MFs), which made it possible to model the correlation between the two parameters in a way that makes sense. It has a triangular, trapezoidal sigmoid and Gaussian member function [15]. Based on the theory, Equations (1) and (2) are created.

(1) $$\mathrm{Rule}\;\mathrm{No}.1:\;\mathrm{if}\;\mu \left(x\right)\;{\mathrm{is}\;A}_{1}\;\mathrm{and}\;\mu \left(y\right)B_{1}{\mathrm{then}\;f}_{1}={\;p}_{1}x+q_{1}y+r_{1}$$

(2) $$\mathrm{Rule}\;\mathrm{No}.\;2:\;\mathrm{if}\;\mu \left(x\right)\;{\mathrm{is}\;A}_{2}\;\mathrm{and}\;\mu \left(y\right){\mathrm{is}\;B}_{2}{\;\mathrm{then}\;f}_{2}={\;p}_{2}x+q_{2}y+r_{2}$$

$A_{1}$,$B_{1},A_{2},B_{2}$ parameters are membership functions for x and y, and inputs $p_{1},q_{1},r_{1},{\;p}_{2},q_{2},r_{2},$ are output function data. The structure of ANFIS and its formulation agrees with a neural net set up with 5 tiers. More details about ANFIS are explained by Khademi et al. 2016.

#### 2.1.3. Generalised Regression Neural Network (GRNN)

GRNN, also known as the lazy training method model, was developed by Specht [16] to behave in the manner of the regression method, by generating a relationship between the dependent manipulated variable (X) and the outcome variable (Y) with a non-linear regression estimation for a smaller group of data. The input layer is similar to that of a conventional neural network, in that its main purpose is to train the input data, and the size of the input vectors is the main determinant of the number of neurons required for training. The model training begins immediately in the pattern layer due to the Gaussian kernel’s conversion of previously input data. The smoothing parameter (*σ*) is used to calculate the weight of each neuron in this layer. This parameter is referred to as the “hyper-parameter of the GRNN model”, and it contributes to the GRNN model’s prediction accuracy [17]. Its general form is depicted as follows.

(3) $$P_{i}=exp\left(-\frac{{\left(X-X_{i}\right)}^{T}\left(X-X_{i}\right)}{2\sigma^{2}}\right)$$

where X equals the input data of the dataset to be tested, $X_{i}$ is the *i*th input of the training dataset, and *σ* is the smoothing parameter.

#### 2.1.4. Interaction Linear Regression (ILR)

Generally, we have different kinds of linear regressions (LRs), including multi-linear regression (MLR), stepwise linear regression (SWLR), and interaction linear regression (ILR), as can be seen from these studies [18,19,20,21].

ILR (isometric log-ratio) is a type of regression that looks at how the relying (target) factors and one or more reaction (response) parameters are related and communicate [22]. Overall, S. I. Abba et al. [23] demonstrates that the multi-linear regression (MLR) concept is the most commonly used regression model. MLR could be read even though it has a lower prediction performance than AI-based modelling techniques [24]. Generally, LR models can be expressed as:

(4) $$y=b_{0}+b_{1}x_{1}+b_{2}x_{2}+\ldots b_{i}x_{i}$$

where *y* represents the target parameter,$\;x_{1}$ equals the value of the 𝑖th predictor, $b_{0}$ denotes constant regression, and $b_{i}$ indicates 𝑖th predictor coefficient.

### 2.2. Hybrid-Based Paradigms

Various modellers have different opinions regarding the buildup of models, methodology employed in inputting data, and duration of modelling; all had a significant impact in optimal performance of the model [25,26,27,28].

The problem associated with artificial intelligence could be surmounted courtesy of evolving techniques utilised in eradicating the issues. This recent outstanding technique takes into consideration the straight line association between the input data and the predicted variable, and also there is no direct relationship between the information and variable it gives out.

These models, in artificial intelligence (AI) models such as Gaussian process regression (GPR), general regression neural networks and adaptive neuro-fuzzy inference, have been impacted positively.

According to the work of Marrero-Ponce et al. [29], ANN (artificial neural network) as well as MLR (multi-linear regression) have been excellent in making remarkable predictions; this is as a result of the combination of rectilinear and haphazard domains of these models and their applications [30,31,32,33]. The “no free lunch” theorem emphatically states that no singular model could be applied to varieties of datasets. The optimal performance of model is dependent on the kind of information the model utilises in working and data features such as measurement of linearity, size, and general wholeness, which all contribute immensely to the working principle of the model.

Several researchers have reiterated that the same information and performance index can vary once various provided variants are utilised.

Again, when data intelligence models are modified, they are wholly utilised in multiple problems.

**Figure 2:**
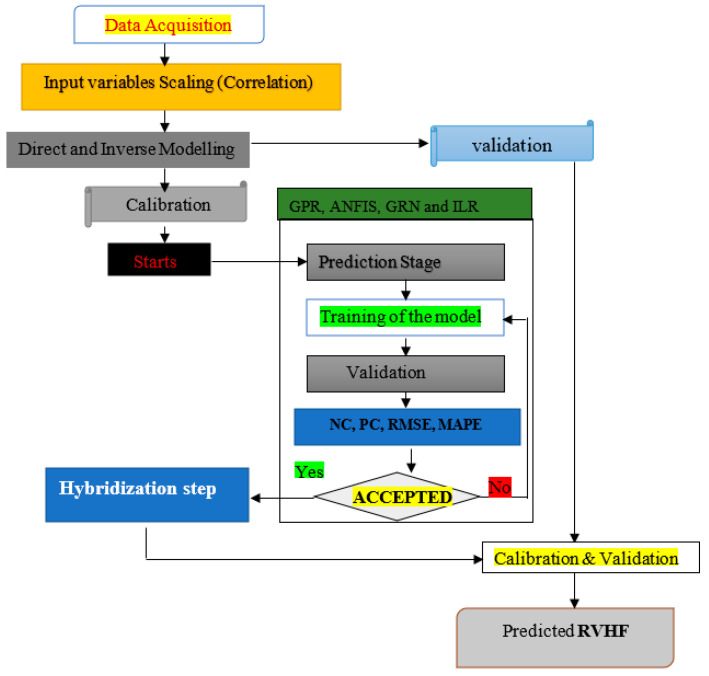
Block diagram depicting the methods used in the current study.

Therefore, in this study, four single models were employed to figure out right ventricular heart failure, which are GPR (Gaussian process regression), GRNN (general regression neural network), adaptive neuro-fuzzy inference, and ILR. An algorithm named “Hybrid Data Intelligence” is then suggested. This combines both the linear ILR model and the various artificial intelligence-based prototypes (ILR, GPR, GRNN, and ANFIS) to reap the benefits of both the unique characteristics and strong points of both models, especially for predicting data trends of various types. The combination of different modelling approaches makes the whole process work efficiently. Afterwards, an ILR model learns in order to acquire the finest models with linear characteristics. The composite (hybrid) method has two categories. ILR, with learning that is associated with general best values, cannot model non-linear features of the data, thereby it returns to the sequential ILR model, which has information about non-linear dynamics and can be utilised by artificial intelligence models to represent information.

(5) $$f\left(y_{t}\right)=q_{t}+r_{t}$$

where $q_{t}$ represents the linear phase, and $r_{t}$ represents the non-linear phase. In order to evaluate how the two proceed, information must be utilised. Let ϵ represent the residual at time *t* from the linear model, therefore:

(6) $$\epsilon_{t}=y_{t}-{\hat{q}}_{t}$$

where ${\hat{q}}_{t}$ is given as forecasted time value t based on the calculated correlation, by modelling residuals using intelligence-based models. It is very vital to find non-linear connections. Using n nodes that provide input, for the residuals, the summation of artificial intelligence model will be:

(7) $$\epsilon_{t}=f\left(\epsilon_{t-1},\epsilon_{t-2},\ldots .,\epsilon_{t-n}\right)+\varepsilon_{t}$$

where *f* is given as the function that is not linear, which was determined by the AI models that make them, GPR, L-Boost, and SVM, giving $\varepsilon_{t}$ as random errors. Moreover, Figure 2 depicts the methods used based on block diagram.

### 2.3. Grading Metrics of the Models Employed in the Current Study

The optimal performance of the model is estimated using different variables that compare estimated values to the one obtained for any category of dataset.

In this work, 2 statistical error metrics were employed, root-mean-square (RMSE) and mean-squared (MSE), plus 2 goodness-of-fit variables: Nash–Sutcliffe coefficient (NC) and Pearson coefficient (PC).

(8) $$\mathrm{NC}=1-\frac{\sum_{j=1}^{N}{\left[{\left(Y\right)}_{obs,j}-{\left(Y\right)}_{com,j}\right]}^{2}}{\sum_{j=1}^{N}{\left[{\left(Y\right)}_{obs,j}-{\bar{\left(Y\right)}}_{obs,j}\right]}^{2}}$$

(9) $$\mathrm{PC}=\frac{\sum_{i=1}^{N}\left(Y_{obs}-{\bar{Y}}_{obs}\right)\left(Y_{com}-{\bar{Y}}_{com}\right)}{\sqrt{\sum_{i=1}^{N}{\left(Y_{obs}-{\bar{Y}}_{obs}\right)}^{2}}\sum_{i=1}^{N}{\left(Y_{com}-{\bar{Y}}_{com}\right)}^{2}\;}\;$$

(10) $$\mathrm{RMSE}=\sqrt{\frac{\sum_{i=1}^{N}{\left(Y_{obsi}-Y_{comi}\right)}^{2}}{N}}$$

(11) $$\mathrm{MSE}=\frac{1}{N}\overset{N}{\underset{i=1}{\sum }}{\left(Y_{obsi}-Y_{comi}\right)}^{2}$$

where *N*, $Y_{obsi}$, $\bar{Y}$, and $Y_{comi}$ are data quantity, data that are seen, overall average of data noted, and calculated values, respectively.

### 2.4. Study Description and Validation Strategy for the Models Used

This study consists of 506 heart-failure subjects from the Faculty of Medicine, Cardiovascular Surgery Department, Ege University, Turkey, who suffered from a severe heart failure and are currently receiving support from a ventricular assistance device. The patients’ group equally consists of RVHF patients, non-RVHF patients, and patients with a risk of RVHF. Whereby, the patients pre-operative features, namely mean pulmonary arterial pressure (mPAP), central venous pressure (CVP), transpulmonary gradient (tpg), preoperative alanine aminotransferase level (ALT), preoperative aspartate aminotransferase level (AST), preoperative billurubin (BUN), prothrombin time (PT time), preoperative haematocrit level (pre htc), preoperative sodium level (pre-sodium), preoperative tricuspid valve insufficiency (pre ty), extracorporeal membrane oxygenator (ECMO), preoperative mitral valve prolapse (preMV), pulmonary capillary wedge pressure (Pcw), preoperative left ventricular end systolic diameter (pre lvesd), preoperative left ventricular ejection fraction (pre lvef), preoperative mitral insufficiency (pre my), preoperative aortic valve insufficiency (pre ay), preoperative systolic pulmonary arterial pressure (pre spap), preoperative tricuspid annular plane systolic excursion (pre tapse), intraaortic balloon pump (IABP), and creatinine were recorded.

Hence, the current study employed the application of both single and hybrid paradigms using different pre-operative variables for the prediction of RVHF. In addition, prior to the modelling step, correlation-based feature extraction step was conducted in order to separate the variables based on their connections with the RVHF output variable. The direct modelling scenario involves pre-operative variables, namely mPAP, CVP, tpg, alt, ast, BUN, pre Bili, PT time, pre htc, pre-sodium, pre ty, ECMO, and preMV for the prediction of RVHF, while the inverse modelling scenario, composed of pcw, creatinine, pre INR, pre lvesd, pre lvef, pre my, pre ay, pre spap, pre tapse, and IABP, was involved in the clinical prediction of RVHF.

Furthermore, the fundamental aim of data-driven techniques is geared toward the model of a collection of datasets, with the pointer in use as a building block for a reliable prediction of unknown. Keeping in mind that several constraints such as overfitting and underfitting lead to poor training and testing results. Testing schemes include k-fold cross-validation, holdout, leave one out, and others. The 10 k-fold cross-validation procedure was used in the current study [34], for assessing and validating the datasets used. According to the k-fold cross-validation, researchers have divided the information: 75% for the calibrating (training) stage and 25% for the checking (verification) stage. Moreover, Table 1 describes the basic descriptive statistics of the study population indicating the mean (0.057312), median (0), mode (0), standard deviation (0.232668), kurtosis (12.64552), skewness (3.820412), range (1), minimum (0), maximum (0), and count (506) of the RVHF patients.

Based on Table 1, the minimum value is 0, which denotes ‘no’, meaning normal patients; the maximum value is 1, meaning ‘yes’, which indicates patients suffering from RVHF.

### 2.5. Model Conceptualisation

Phase 1: Data acquisition

The complete dataset was obtained from a clinical study consisting of 506 heart-failure subjects from the Faculty of Medicine, Cardiovascular Surgery Department, Ege University, Turkey, who suffered from a severe heart failure and are currently receiving support from a ventricular assistance device. Furthermore, the data points were divided into 75% for the calibration stage and 25% for the testing stage. The data were subsequently validated to check and control potential modelling problems such as overfitting and underfitting.

Phase 2: Simulation using single models

The stand-alone paradigms (GPR, GRNN, and ANFIS) together with the traditional linear regression ILR were all conducted using MATLAB 9.3 (R2020a).

Phase 3: Hybrid data-intelligence

The data-intelligence techniques involve combining the properties of the classical linear ILR technique with AI-based techniques in order to improve the performance of the single models. Hence, ILR, GPR, GRNN, and ANFIS are developed.

Furthermore, the major essence of developing these techniques is to understand the behaviour of different clinical data (consisting of the input and output variables) to assign some weight that can be used in predicting the target. For instance, in the current study, the pre-operative variables are used in determining whether a patient will suffer from RVHF or not. Moreover, developing mobile application is very possible through this approach, in order to assist clinicians, patients, and policy makers in understanding the behaviour and risk factors of RVHF.

## 3. Results

Recently, computational techniques have been established in the medical field for the prediction of various diseases, prevalence, and disorders. AI-based techniques and machine learning are the major dominant techniques over classical regressions, owing to their robustness in handling highly chaotic datasets. Therefore, understanding a dataset prior to the employment of AI techniques for prediction is of paramount importance. Different feature-extraction techniques such as mutual understanding, sensitivity analysis, and correlational analysis feature the extraction method. Therefore, an exploratory method based on correlation analysis is utilised in Table 2 in order to determine the input–output relation of the variables used in the current study.

An exploratory technique informed of correlation analysis is employed in Table 2 to elucidate the relation between the variables used in the current study.

### Performance of the Single and Hybrid Paradigms for Modelling RVHF Using Pre-Operative Variables

The currect section demonstrates both the quantitative and visualised performance of both the single and hybrid paradigms for the direct and inverse prediction of RVHF using various pre-operative variables.

## 4. Discussion

The correlational analysis shown in Table 2 demonstrates both the direct and inverse relation for the input and the corresponding RVHF variable. Based on Table 2a, all the independent variables showed a weak connection with the output variable (RVHF), whereby pre sodium and PT time showed a superior relation than the others, with R-values equal to 0.16 and 0.15, respectively. Moreover, CVP showed a 0 correlational value with RVHF; this is due to the fact that most of the patients are suffering from left ventricular heart failure but not RVHF. Furthermore, Table 2b equally indicates that all the input variables showed a weak negative correlation with RVHF as the corresponding output variable. Based on the inverse results, pre my with an R-value equal to −0.24 with RVHF showed the highest relation with the output variable, in which pre spap showed a 0 relation with the corresponding RVHF output variable.

Based on the correlational analysis performance shown in Table 2, four different single-paradigm (GPR, GRNN, ANFIS, and ILR) models integrated with three different novel hybrid paradigms (ILR–GPR, ILR–GRNN, and ILR–ANFIS) were used in predicting RVHF using various pre-operative variables in two different scenarios (direct and inverse approaches).

Table 3 demonstrates the performance of the direct modelling scenario approach for the prediction of RVHF. The performance metrics, PC, DC, RMSE, and MSE, used in the current scenario indicate the superiority of the hybrid techniques over the single approaches for the clinical modelling of RVHF in both the calibration and validation stages. Various modellers reported that for any prediction model or computation paradigm to be accepted, it should show a minimum DC-value of 0.8. Therefore, all four single models (ILR, ANFIS, GRNN, and GPR) failed to fulfill this criterion, as they failed to meet this requirement in both the calibration and validation phases. In addition, ILR–GPR and ILR–ANFIS, with DC-values equal to 0.862 and 0.813, respectively, in the calibration phase fulfilled the minimum requirement. Whereby, ILR–ANFIS (0.696) failed and ILR–GPR (0.861) succeeded in the validation step. The weak performance of the paradigms can be attributed to the low correlation between the pre-operative variables and RVHF as the corresponding output variable, as shown in Table 2a. In general, for both the single and hybrid paradigms used in the direct modelling of RVHF based on the pre-operative variables, only the ILR–GPR hybrid technique was able to capture the highly non-linear and chaotic nature of the dataset.

Hence, the performance of the results obtained in the current study is in line with studies reported by Konstantinos et al. [35] regarding the current and future state of the AI-enhanced electrocardiogram in detecting heart disease for patients in at-risk population densities. Their technique has made it possible for the electrocardiogram to be interpreted quickly.

Moreover, Muni et al. used AI techniques to present the importance of passive and semi-sensors and unique approaches analysing heart failure [36]. More studies related with the implementation of ML and AI on cardiovascular diseases require elucidation.

**Figure 3:**
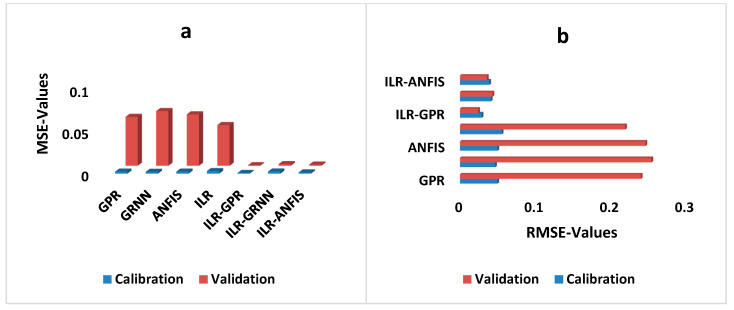
Error-depicted interims of (**a**) MSE- and (**b**) RMSE-values for the direct modelling of both the single and hybrid paradigms.

The performance of both the single and hybrid techniques can be comparatively visualised using various graphical illustrations. For instance, the MSE- and RMSE-values are used to indicate the error performance of each model. The error performance of both the single and hybrid techniques developed using the direct modelling approach can be graphically compared in both the calibration and validation steps using column and bar charts (see Figure 3).

**Figure 4:**
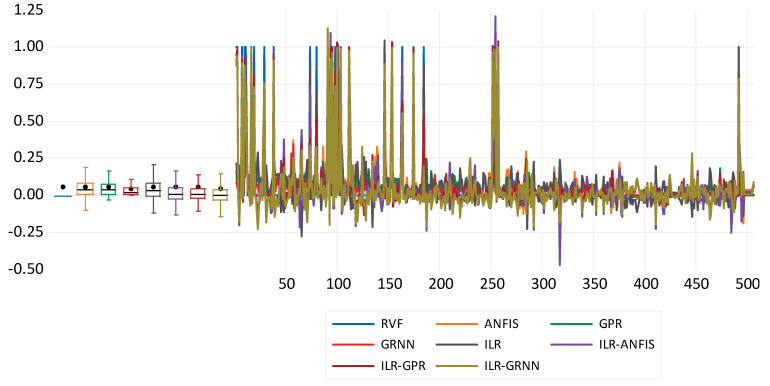
Response plot for the direct modelling approach of right ventricular heart failure for both the single and hybrid approaches.

Moreover, the fitness of the direct modelling can be compared based on performances against the clinical RVHF values, which can be visualised using a time series plot (see Figure 4).

**Figure 5:**
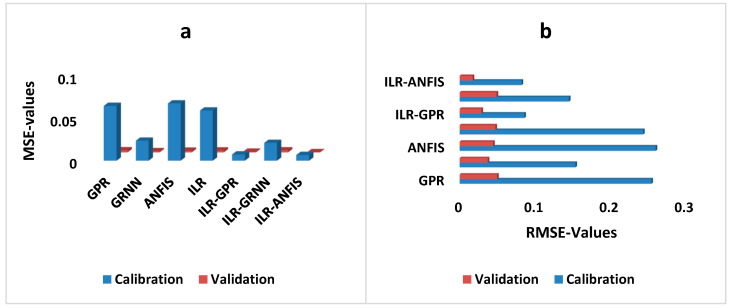
Error-depicted interims of (**a**) MSE- and (**b**) RMSE-values for the direct modelling of both the single and hybrid paradigms.

Furthermore, Table 4 indicates the performance of inverse modelling for the prediction of RVHF in both the calibration and validation steps. Based on the quantitative performance of the single and hybrid techniques, it can be seen that all four single models, GPR, GRNN, ANFIS, and ILR, failed to predict RVHF as the dependent variable. Whereas, for the hybrid-based inverse modelling, ILR–GPR and ILR–ANFIS were able to predict the behaviour and properties of the complex RVHF dataset. Moreover, comparative analysis of all the inverse-based techniques indicated that ILR–ANFIS outperformed all six other techniques in both the training and validation stages. Furthermore, based on the DC-metrics performance of the ILR–ANFIS technique, its ability in improving the performance prediction of the single paradigms increased up to 81% and 51% in the calibration and validation stages, respectively. Hence, the comparative performance of the techniques can be graphically compared, based on the performance-error in terms of RMSE and MSE (see Figure 5).

**Figure 6:**
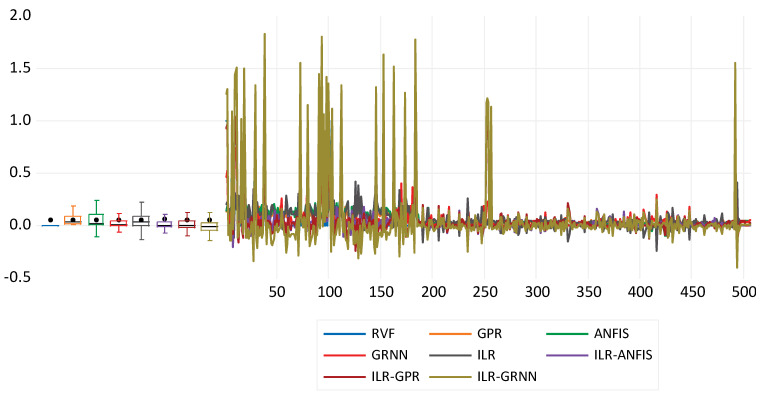
Response plot for the inverse modelling approach of right ventricular heart failure for both the single and hybrid approaches.

Moreover, the metrics PC and DC indicate the performance fitness between the predicted and experimental values. Therefore, the response plot information of the time series can be used to compare the performance of the hybrid techniques for the simulation of RVHF (see Figure 6).

Hence, the novelty of the current work can be shown in different ways: (1) This is the first study that reports the combined application of GPR, GRNN, and ANFIS AI-based techniques for the clinical prediction of RVHF using pre-operative variables. (2) It is, equally, the first study that employs the application of the ILR regression method for the clinical modelling of RVHF; in fact, this is the first study that reports the implementation of this model in any clinical/health-related study. Ultimately, to the best knowledge of the authors, based on the recent technical literature as well as a scan of the literature, as shown in Figure 1, this is the first study that reports the feasibility of applying direct and inverse modelling for clinical prediction in health- and medical-related studies. Moreover, the quantitative performance of the hybrid technique based on the Nash–Sutcliffe coefficient (NC) metric depicts its superiority over the single paradigms by up to 58.7%/75.5% and 80.3%/51% for the calibration/validation phases in the direct and inverse modelling approaches, respectively. However, one of the major limitations of the current study is the employment of a two-step technique: the single and hybrid approaches; whereby, the single approach was unable to capture the RVHF datasets owing to its complexity and chaotic nature.

## 5. Conclusions

Medical informatics deals with improving the management of clinical knowledge, patient data, population data, and information related to patient care. This emerging technique is regarded as a promising tool that helps policy and decision makers in making critical decisions related to patients’ care. Therefore, the current study explores the application of both direct and inverse modelling using AI-based techniques and hybrid-based paradigms for the prediction of RVHF; whereby, the hybrid techniques depict a higher performance compared with the single paradigms. Hence, the results of the current research recommend the application of various metaheuristic and computational approaches for improving the prediction ability of RVHF using various pre-operative variables. Furthermore, future work on different ways of identifying the complex behaviour of the data through the non-linear feature extraction technique, feature scaling, the normalisation of data, and standardisation are equally recommended.

## Figures and Tables

**Table 1:** Descriptive analysis of the study population.

Operation	RVHF
Mean	0.057312
Median	0
Mode	0
Standard Deviation	0.232668
Kurtosis	12.64552
Skewness	3.820412
Range	1
Minimum	0
Maximum	1
Count	506

**Table 2:** (**a**) Direct and (**b**) inverse correlation analysis.

**(a) Direct**														
**Variables**	**mPAP**	**CVP**	**tpg**	**alt**	**ast**	**BUN**	**pre Bili**	**PT time**	**pre htc**	**pre sodium**	**pre ty**	**ECMO**	**preMV**	**RVHF**
**mPAP**	1													
**CVP**	0.42	1.00												
**tpg**	0.51	0.48	1.00											
**alt**	−0.05	−0.04	−0.08	1.00										
**ast**	0.01	0.03	0.06	0.41	1.00									
**BUN**	−0.05	−0.03	0.03	0.10	0.20	1.00								
**pre Bilirubin**	0.08	0.32	0.16	−0.03	0.25	0.07	1.00							
**PT time**	0.06	0.02	0.09	0.11	0.00	0.07	0.04	1.00						
**pre htc**	0.03	−0.04	0.10	0.03	−0.07	0.05	−0.12	0.17	1.00					
**pre sodium**	−0.12	−0.09	−0.05	0.09	−0.01	0.05	−0.21	0.20	0.21	1.00				
**pre ty**	0.06	0.19	0.13	−0.02	0.21	0.07	0.56	0.06	−0.07	−0.09	1.00			
**ECMO**	−0.07	−0.06	0.00	0.04	0.48	0.14	0.34	−0.05	−0.08	0.02	0.20	1.00		
**preMV**	−0.03	0.14	−0.13	0.06	0.07	−0.08	0.21	−0.02	−0.13	0.00	0.11	0.03	1.00	
**RVHF**	0.02	0.00	0.05	0.03	0.07	0.07	0.01	0.15	0.01	0.16	0.02	0.10	0.01	1
**(b) Inverse**														
**Variables**	**pcw**		**creatinin**		**pre INR**		**pre lvesd**	**pre lvef**	**pre my**	**pre ay**	**pre spap**	**pre tapse**	**IABP**	**RVHF**
**pcw**	1													
**creatinin**	0.14		1.00											
**pre INR**	0.16		0.60		1.00									
**pre lvesd**	0.11		0.38		0.79		1.00							
**pre lvef**	0.07		0.09		−0.16		−0.33	1.00						
**pre my**	0.05		0.08		0.00		−0.11	0.21	1.00					
**pre ay**	0.12		0.30		0.58		0.74	−0.28	−0.05	1.00				
**pre spap**	0.16		−0.17		−0.27		−0.28	0.20	0.08	−0.20	1.00			
**pre tapse**	0.01		−0.16		−0.28		−0.32	0.24	0.05	−0.22	0.25	1.00		
**IABP**	0.10		0.31		0.66		0.83	−0.37	−0.10	0.80	−0.28	−0.27	1.00	
**RVHF**	−0.03		−0.04		−0.04		−0.03	−0.03	−0.24	−0.02	0.00	−0.01	−0.03	1.00

**Table 3:** Performance of the direct modelling scenario approach.

**Calibration**				
**Models**	**DC**	**PC**	**MSE**	**RMSE**
**GPR**	0.419	0.647	0.002	0.050
**GRNN**	0.485	0.697	0.002	0.047
**ANFIS**	0.416	0.645	0.003	0.050
**ILR**	0.275	0.525	0.003	0.056
**ILR–GPR**	0.862	0.928	0.001	0.029
**ILR–GRNN**	0.777	0.882	0.003	0.041
**ILR–ANFIS**	0.813	0.902	0.001	0.039
**Validation**				
**GPR**	0.201	0.449	0.058	0.241
**GRNN**	0.106	0.325	0.065	0.255
**ANFIS**	0.160	0.400	0.061	0.247
**ILR**	0.335	0.578	0.048	0.220
**ILR–GPR**	0.861	0.928	0.001	0.024
**ILR–GRNN**	0.559	0.748	0.002	0.044
**ILR–ANFIS**	0.696	0.834	0.001	0.036

**Table 4:** Performance of the inverse modelling scenario approach.

**Calibration**				
	**DC**	**PC**	**MSE**	**RMSE**
**GPR**	0.103	0.321	0.065	0.255
**GRNN**	0.673	0.820	0.024	0.154
**ANFIS**	0.063	0.251	0.068	0.261
**ILR**	0.179	0.423	0.060	0.244
**ILR–GPR**	0.898	0.947	0.007	0.086
**ILR–GRNN**	0.709	0.842	0.021	0.146
**ILR–ANFIS**	0.906	0.952	0.007	0.082
**Validation**				
**GPR**	0.419	0.647	0.002	0.050
**GRNN**	0.673	0.820	0.001	0.037
**ANFIS**	0.537	0.733	0.002	0.045
**ILR**	0.472	0.687	0.002	0.048
**ILR–GPR**	0.802	0.896	0.001	0.029
**ILR–GRNN**	0.437	0.661	0.002	0.049
**ILR–ANFIS**	0.929	0.964	0.000	0.017

## Data Availability

Data will be provided on requests.

## References

[1] Sztrymf B., Vuillard C., Boucly A., Artaud-Macari E., Sattler C., Amar D., Jaïs X., Sitbon O., Humbert M., Savale L. (2022). Right heart failure. ERS Monogr..

[2] Andrade L., Carazo M., Wu F., Kim Y., Wilson W. (2019). Mechanisms for heart failure in systemic right ventricle. Heart Fail. Rev..

[3] Wang J.M., Rai R., Carrasco M., Sam-Odusina T., Salandy S., Gielecki J., Zurada A., Loukas M. (2020). An anatomical review of the right ventricle. Transl. Res. Anat..

[4] Pfeffer M.A., Shah A.M., Borlaug B. (2019). Heart Failure With Preserved Ejection Fraction In Perspective. Circ. Res..

[5] Grünig E., Eichstaedt C.A., Seeger R., Benjamin N. (2020). Right Heart Size and Right Ventricular Reserve in Pulmonary Hypertension: Impact on Management and Prognosis. Diagnostics.

[6] Ji M., Wu W., He L., Gao L., Zhang Y., Lin Y., Qian M., Wang J., Zhang L., Xie M. (2022). Right Ventricular Longitudinal Strain in Patients with Heart Failure. Diagnostics.

[7] Zhang X., Ruan B., Qiao Z., Yang M., Zhuang Q., Wang J., Wang W., Zheng Y., Zhao H., Shen X. (2022). The Balance between the Left and Right Ventricular Deformation Evaluated by Speckle Tracking Echocardiography Is a Great Predictor of the Major Adverse Cardiac Event in Patients with Pulmonary Hypertension. Diagnostics.

[8] Manca P., Cannatà A., Nuzzi V., Bromage D.I., Varrà G.G., Rossi M., Ferro M.D., Paldino A., Gigli M., Barbati G. (2021). Prevalence and Evolution of Right Ventricular Dysfunction Among Different Genetic Backgrounds in Dilated Cardiomyopathy. Can. J. Cardiol..

[9] Patil S., Albogami S., Hosmani J., Mujoo S., Kamil M.A., Mansour M.A., Abdul H.N., Bhandi S., Ahmed S.S.S.J. (2022). Artificial Intelligence in the Diagnosis of Oral Diseases: Applications and Pitfalls. Diagnostics.

[10] Lv J., Bin Dong B., Lei H., Shi G., Wang H., Zhu F., Wen C., Zhang Q., Fu L., Gu X. (2021). Artificial intelligence-assisted auscultation in detecting congenital heart disease. Eur. Heart J.-Digit. Health.

[11] Romiti S., Vinciguerra M., Saade W., Cortajarena I.A., Greco E. (2020). Artificial Intelligence (AI) and Cardiovascular Diseases: An Unexpected Alliance. Cardiol. Res. Pract..

[12] Omran B.A., Chen Q., Jin R. (2016). Comparison of Data Mining Techniques for Predicting Compressive Strength of Environmentally Friendly Concrete. J. Comput. Civ. Eng..

[13] Cheng M.-Y., Huang C.-C., Van Roy A.F. (2013). Predicting project success in construction using an evolutionary gaussian process inference model. J. Civ. Eng. Manag..

[14] Pal M., Deswal S. (2010). Modelling pile capacity using Gaussian process regression. Comput. Geotech..

[15] Elkiran G., Nourani V., Abba S. (2019). Multi-step ahead modelling of river water quality parameters using ensemble artificial intelligence-based approach. J. Hydrol..

[16] Wiangkham A., Ariyarit A., Aengchuan P. (2022). Prediction of the influence of loading rate and sugarcane leaves concentration on fracture toughness of sugarcane leaves and epoxy composite using artificial intelligence. Theor. Appl. Fract. Mech..

[17] Ardejanii F.D., Rooki R., Shokri B.J., Kish T.E., Aryafar A., Tourani P. (2013). Prediction of Rare Earth Elements in Neutral Alkaline Mine Drainage from Razi Coal Mine, Golestan Province, Northeast Iran, Using General Regression Neural Network. J. Environ. Eng..

[18] Usman A.G., Işik S., Abba S.I. (2021). Hybrid data-intelligence algorithms for the simulation of thymoquinone in HPLC method development. J. Iran. Chem. Soc..

[19] Abba S.I., Abdulkadir R.A., Sammen S.S., Usman A.G., Meshram S.G., Malik A., Shahid S. (2021). Comparative implementation between neuro-emotional genetic algorithm and novel ensemble computing techniques for modelling dissolved oxygen concentration. Hydrol. Sci. J..

[20] Ghali U.M., Usman A.G., Chellube Z.M., Degm M.A.A., Hoti K., Umar H., Abba S.I. (2020). Advanced chromatographic technique for performance simulation of anti-Alzheimer agent: An ensemble machine learning approach. SN Appl. Sci..

[21] Usman A.G., Işik S., Abba S.I., Meriçli F. (2021). Chemometrics-based models hyphenated with ensemble machine learning for retention time simulation of isoquercitrin in Coriander sativum L. using high-performance liquid chromatography. J. Sep. Sci..

[22] Asadisaghandi J., Tahmasebi P. (2011). Comparative evaluation of back-propagation neural network learning algorithms and empirical correlations for prediction of oil PVT properties in Iran oilfields. J. Pet. Sci. Eng..

[23] Abba S., Usman A., Işik S. (2020). Simulation for response surface in the HPLC optimization method development using artificial intelligence models: A data-driven approach. Chemom. Intell. Lab. Syst..

[24] Nemati S., Fazelifard M.H., Terzi Ö., Ghorbani M.A. (2015). Estimation of dissolved oxygen using data-driven techniques in the Tai Po River, Hong Kong. Environ. Earth Sci..

[25] Garba Usman A., Alhosen M., Alsharksi A., Muhammed Naibi A. Applications of Artificial Intelligence-Based Models and Multi-Linear Regression for the Prediction of Thyroid Stimulating Hormone Level in the Human Body Artificial Intelligent Techniques for Stream Flow Modeling View Project Global Platform to Showcase Your Research View project. https://www.researchgate.net/publication/342571024.

[26] Usman A.G., Işik S., Abba S.I., Meriçli F. (2020). Artificial intelligence–based models for the qualitative and quantitative prediction of a phytochemical compound using HPLC method. Turk. J. Chem..

[27] Modeling of Water Treatment Plant Performance Using Artificial Neural Network: Case Study Tamburawa Kano-Nigeria. https://www.researchgate.net/publication/344380629_Modeling_of_Water_Treatment_Plant_Performance_using_Artificial_Neural_Network_Case_Study_Tamburawa_Kano-Nigeria.

[28] Gaya M.S., Abba S.I., Abdu A.M., Tukur A.I., Saleh M.A., Esmaili P., Wahab N.A. (2020). Estimation of water quality index using artificial intelligence approaches and multi-linear regression. IAES Int. J. Artif. Intell. (IJ-AI).

[29] Marrero-Ponce Y., Barigye S.J., Jorge-Rodríguez M.E., Tran-Thi-Thu T. (2018). QSRR prediction of gas chromatography retention indices of essential oil components. Chem. Pap..

[30] Malami S.I., Musa A.A., Haruna S.I., Aliyu U.U., Usman A.G., Abdurrahman M.I., Bashir A., Abba S.I. (2021). Implementation of soft-computing models for prediction of flexural strength of pervious concrete hybridized with rice husk ash and calcium carbide waste. Model. Earth Syst. Environ..

[31] Alamrouni A., Aslanova F., Mati S., Maccido H.S., Jibril A.A., Usman A.G., Abba S.I. (2022). Multi-Regional Modeling of Cumulative COVID-19 Cases Integrated with Environmental Forest Knowledge Estimation: A Deep Learning Ensemble Approach. Int. J. Environ. Res. Public Health.

[32] Khalid G.M., Usman A.G. (2021). Application of data-intelligence algorithms for modeling the compaction performance of new pharmaceutical excipients. Future J. Pharm. Sci..

[33] Haruna S.I., Malami S.I., Adamu M., Usman A.G., Farouk A., Ali S.I.A., Abba S.I. (2021). Compressive Strength of Self-Compacting Concrete Modified with Rice Husk Ash and Calcium Carbide Waste Modeling: A Feasibility of Emerging Emotional Intelligent Model (EANN) Versus Traditional FFNN. Arab. J. Sci. Eng..

[34] Usman A.G., Işik S., Abba S.I. (2020). A Novel Multi-model Data-Driven Ensemble Technique for the Prediction of Retention Factor in HPLC Method Development. Chromatographia.

[35] Siontis K.C., Noseworthy P.A., Attia Z.I., Friedman P.A. (2021). Artificial intelligence-enhanced electrocardiography in cardiovascular disease management. Nat. Rev. Cardiol..

[36] Maurya M.R., Riyaz N.U.S.S., Reddy M.S.B., Yalcin H.C., Ouakad H.M., Bahadur I., Al-Maadeed S., Sadasivuni K.K. (2021). A review of smart sensors coupled with Internet of Things and Artificial Intelligence approach for heart failure monitoring. Med. Biol. Eng. Comput..

